# Automated video recording built into routine clinical practice: What does it take?

**DOI:** 10.1055/a-2828-9572

**Published:** 2026-03-24

**Authors:** Cadman Leggett, Jeffrey Fetzer, John League, Shounak Majumder, Darrell Pardi, Nayantara Coelho-Prabhu

**Affiliations:** 1 Division of Gastroenterology and Hepatology 6915 Mayo ClinicRochesterMinnesotaUnited States



We read with great interest the article “Implementing endoscopy video recording in routine clinical practice: Strategies from three tertiary care centers” and commend the authors for highlighting diverse approaches to integrating video capture into endoscopy workflows 1 . In the spirit of contributing to this important discussion, we would like to share our experience at Mayo Clinic Rochester (MCR), where endoscopy video recording has become a standard part of clinical practice.

MCR operates 27 endoscopy rooms equipped with Olympus and Fujifilm video processors. For video capture, we have implemented the Sony NUCLeUS video-over-IP platform, deployed entirely on-premises and recording in 1080p. This architecture was chosen specifically to address concerns around data ownership and security that are often associated with commercial cloud-based solutions. The infrastructure needs included a Data Scientist, HTM & IT support staff, high-speed network area storage, local area network with layer 3 network switch to support Virtual local area network(s) and multicasting, electronic health record (EHR) interfaces, and dedicated rack space in a data center or closet for the servers.

Our system is distinguished by a custom-built, artificial intelligence-driven automation layer that interfaces with the NUCLeUS API. This solution manages the entire video workflow—from referencing the hospital worklist to initiating and terminating recordings, and post-processing the videos (including trimming, redacting, indexing, and labeling). The core of our automation is a system built upon a foundation model and pretrained on a large and diverse dataset of endoscopy videos, which provides contextual understanding of procedures, including scope in/out detection, and supports advanced procedures such as endoscopic retrograde cholangiopancreatography and endoscopic ultrasound.

NUCLeUS also supports multi-stream recording, enabling simultaneous capture of fluoroscopy and endoscopy feeds. Once recorded, videos are automatically linked to our internal video repository, VideoHub+, which is hosted on HITrust-compliant, private, non-proprietary servers within our internal cloud. This repository integrates with the EHR, allowing linkage to procedure reports, histology results, and the longitudinal patient record ( Fig. 1 ). Endoscopists retain control over their content, with permissions to view, download, or edit videos. Usage tracking further enhances data governance and patient privacy. The system is also integrated with existing infrastructure such as the Stryker SDC3, ensuring seamless deployment.

**Fig. 1:**
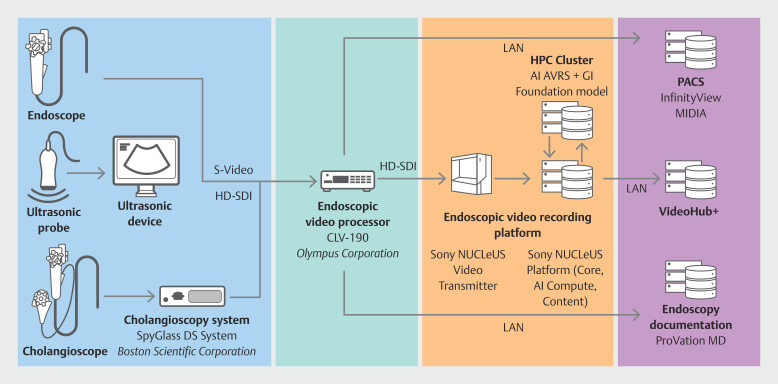
Graphic displaying auto video recording at Mayo Clinic Rochester. Source: Steinhäuser JL, Berzin TM, Feissler ME et al. Implementing endoscopy video recording in routine clinical practice: Strategies from three tertiary care centers. Endosc Int Open 2025; 13: a25923338. 10.1055/a-2592–3338. © 2025. The Author(s). Licensed under Creative Commons Attribution License (CC BY) https://creativecommons.org/licenses/by/4.0/ . Modifications applied.

Currently, our implementation contributes over 100 endoscopy videos daily to our database. This model represents both a synthesis and an evolution of the approaches described in the original article. It combines the institutional control and customizability of the Würzburg system with a higher degree of automation than the commercial solution used in Boston, all while maintaining strict on-premises data security.

We believe the Mayo Clinic framework offers a scalable, secure, and highly automated model for endoscopy video recording that can be adapted by other institutions seeking to enhance clinical documentation, quality assurance, and research capabilities.

**Publication note**

Letters to the editor do not necessarily represent the opinion of the editor or publisher. The editor and publisher reserve the right to not publish letters to the editor, or to publish them abbreviated or in extracts.

## Notes

Correction **Automated video recording built into routine clinical practice: What does it take?** Cadman Leggett, Jeffrey Fetzer, John League et al. Endoscopy International Open 2026; 14: a28289572. DOI: 10.1055/a-2828-9572 In the above-mentioned article figure 1 was corrected. This was corrected in the online version on 24 April 2026.
