# Correction: Automated video recording built into routine clinical practice: What does it take?

**DOI:** 10.1055/a-2860-7700

**Published:** 2026-04-24

**Authors:** Cadman Leggett, Jeffrey Fetzer, John League, Shounak Majumder, Darrell Pardi, Nayantara Coelho-Prabhu

**Affiliations:** 1 Division of Gastroenterology and Hepatology 6915 Mayo ClinicRochesterMinnesotaUnited States



In the above-mentioned article figure 1 was corrected. This was corrected in the online version on 24 April 2026.

